# Modified Back Contact Interface of CZTSe Thin Film Solar Cells: Elimination of Double Layer Distribution in Absorber Layer

**DOI:** 10.1002/advs.201700645

**Published:** 2017-11-20

**Authors:** Zhaojing Zhang, Liyong Yao, Yi Zhang, Jianping Ao, Jinlian Bi, Shoushuai Gao, Qing Gao, Ming‐Jer Jeng, Guozhong Sun, Zhiqiang Zhou, Qing He, Yun Sun

**Affiliations:** ^1^ Institute of Photoelectronic Thin Film Devices and Technology Tianjin Key Laboratory of Thin film Devices and Technology Nankai University Tianjin 300071 P. R. China; ^2^ Tianjin Institute of Power Source Tianjin 300384 P. R. China; ^3^ Department of Electronic Engineering Chang Gung University Taoyuan 33302 Taiwan

**Keywords:** CZTSe, double layer distribution, kesterite, solar cells

## Abstract

Double layer distribution exists in Cu_2_SnZnSe_4_ (CZTSe) thin films prepared by selenizing the metallic precursors, which will degrade the back contact of Mo substrate to absorber layer and thus suppressing the performance of solar cell. In this work, the double‐layer distribution of CZTSe film is eliminated entirely and the formation of MoSe_2_ interfacial layer is inhibited successfully. CZTSe film is prepared by selenizing the precursor deposited by electrodeposition method under Se and SnSe*_x_* mixed atmosphere. It is found that the insufficient reaction between ZnSe and Cu‐Sn‐Se phases in the bottom of the film is the reason why the double layer distribution of CZTSe film is formed. By increasing Sn content in the metallic precursor, thus making up the loss of Sn because of the decomposition of CZTSe and facilitate the diffusion of liquid Cu_2_Se, the double layer distribution is eliminated entirely. The crystallization of the formed thin film is dense and the grains go through the entire film without voids. And there is no obvious MoSe_2_ layer formed between CZTSe and Mo. As a consequence, the series resistance of the solar cell reduces significantly to 0.14 Ω cm^2^ and a CZTSe solar cell with efficiency of 7.2% is fabricated.

## 1 Introduction

Cu‐based semiconductor materials Cu_2_SnZnS_4_ (CZTS), Cu_2_SnZnSe_4_ (CZTSe), and Cu_2_ZnSn(S,Se)_4_ (CZTSSe) have attracted increasing attention because of its reasonable photoelectric performance and earth‐abundant constituent elements compared with the traditional Cu(In,Ga)Se_2_ (CIGSe) material. Up to now, an excellent power conversion efficiency of 12.6% for CZTSSe solar cell has been achieved based on hydrazine solution method.^1^ However, the development of these absorber materials still has a huge distance from CIGSe. The most important reason for this issue should be contributed to the large *V*_oc_ deficit, which is related to many aspects, such as defects, absorber crystallization, and homogeneous composition. Among these aspects, quality of absorber layer and back contact are very important for the performance of CZTSe solar cells. It is general that the absorber layer is double layer distribution as it is prepared by selenizing metallic precursors. Large grains are presented in the up layer, whereas small grains are appeared in the bottom layer of the film.^2^, ^3^, ^4^, ^5^, ^6^ The smaller grains lead to more grain boundaries which can provide a leakage pathway by having photogenerated electrons flow toward the back contact, thus deteriorate the device efficiency. However, it should be noted that very little work has been done on this phenomenon. J. J. Scragg found that there is aggressive reaction between CZTS absorber and Mo substrate, which cause the formation of MoS_2_ and other secondary phases during annealing process.^7^ As a result, the phenomenon of double layer distribution appears in the bottom of the film. It seems that this phenomenon cannot be eliminated unless the Mo substrate is replaced. Recently, Jong‐Ok Jeon carried out the selenization at different thermal annealing temperature and largely eliminated the double layer distribution. However, a thick MoSe_2_ layer between Mo and absorber layer was formed and a lot of voids were observed in CZTSe film at the same time.^8^ It has been indicated that thick MoSe_2_ will deteriorate device performance significantly by increasing the series resistance of solar cells.^9^, ^10^ Therefore, the elimination of double layer distribution and suppressing the MoSe_2_ formation are crucial for the development of CZTSe thin film solar cell.

In this study, we prepared the CZTSe film by selenizing the metallic precursor prepared by electrodeposition method in Se and SnSe*_x_* atmosphere. We find that the high content of Zn element in the bottom of the film is the reason for the formation of double layer distribution in CZTSe film. Based on this finding, double layer distribution was eliminated by controlling the composition of metallic precursor, and no MoSe_2_ layer was formed. The short‐circuit photocurrent density (*J*_sc_) was increased from 30.4 to 37.6 mA cm^−2^ and the series resistance was reduced significantly to 0.14 Ω cm^2^ as the double layer distribution of CZTSe film was eliminated. As a result, the performance of CZTSe solar cells is improved significantly by restraining the formation of double layer distribution.

## 2 Results and Discussion

### 2.1 Elimination of Double Layer Distribution

**Figure 1:**
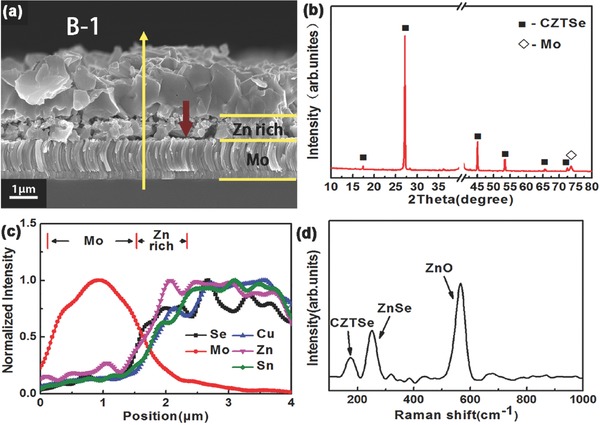
a) Cross‐section SEM image, b) XRD spectra, c) EDS depth profile, and d) Raman spectra of sample B‐1. The Raman spectra taken at the back surface of the absorber. The red arrow in (a) points the position of the incident laser.

The preparation process of CZTSe film is shown in Figure S1 (Supporting Information). **Table** **1** lists the compositions of precursor (sample A‐1) after preheating process (sample A‐1‐h) followed by preselenizing process (sample A‐1‐hs) and low‐pressure selenization process (sample B‐1). All the samples are Cu‐poor and Zn‐rich. Cross‐section morphology image and energy‐dispersive spectrometer (EDS) depth profile of the synthesized CZTSe thin film (sample B‐1) are shown in **Figure** **1**a,c. It can be clearly seen that the grains of the film exhibit a distinct double layer distribution: the up layer is composed of large and compact grains, and the bottom layer is composed of small and loose grains. No obvious MoSe_2_ layer is observed. As shown in Figure 1c, the signal intensities of Cu and Sn elements almost decrease with the same slope from surface to bottom of sample B‐1, while Zn signal is basically unchanged from surface to bottom. Considering the small voids at the bottom of the film, it can be inferred that the bottom layer composed of small grains is Zn‐richer than the upper layer. X‐ray diffraction (XRD) pattern of sample B‐1, shown in Figure 1b, indicates that the film is composed of CZTSe or ZnSe. It is difficult to figure out the ZnSe secondary phase by XRD because ZnSe reflections are overlapped by the reflections of CZTSe. To make clear whether there exists ZnSe phase at the bottom Zn‐rich region, Raman spectra (Figure 1d) were directly measured on the bottom of sample B‐1 using 325 nm laser as the exciting source (after mechanical removal of the absorber from substrate). Obviously a peak centered at 252 cm^−1^ attributed to ZnSe is observed.^11^ Additionally, the ZnO peak centered at ≈575 cm^−1^ can also be observed,^12^ which should be the result of the introduction of oxygen element during precursors prepared by electrodeposition process. Raman results illustrate that the excess Zn element in the bottom of the film is mainly presented in the form of ZnSe phase. The insufficient diffusion of Zn element and the inadequate reaction of ZnSe phase in the bottom layer may attribute to the phenomenon of double layer distribution.

**Table 1:** The compositions of samples A‐1, A‐1‐h, A‐1‐hs, A‐2, A‐3, B‐1, B‐2, and B‐3 which were measured by XRF. M = Cu + Sn + Zn + Se, where Cu, Zn, Sn, and Se are the atomic fractions

Samples	Type	Cu/M	Zn/M	Sn/M	Se/M	Zn/Sn
A‐1	Metallic precursor	0.40	0.33	0.27	0	1.22
A‐1‐h	Sample A‐1 after preheating process	0.40	0.33	0.27	0	1.22
A‐1‐hs	Sample A‐1‐h after preselenizing process	0.23	0.17	0.14	0.46	1.21
A‐2	Metallic precursor	0.38	0.31	0.31	0	1.0
A‐3	Metallic precursor	0.34	0.29	0.37	0	0.78
B‐1	Sample A‐1‐hs after selenization process	0.21	0.16	0.12	0.51	1.32
B‐2	Sample A‐2 after all process	0.21	0.15	0.12	0.51	1.25
B‐3	Sample A‐3 after all process	0.22	0.15	0.11	0.52	1.36

**Figure 2:**
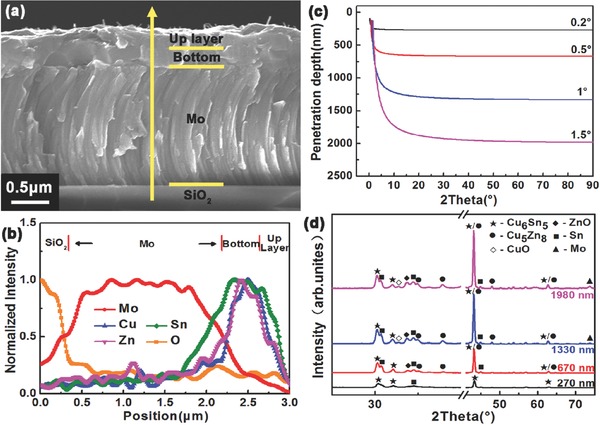
The a) cross‐section morphology and b) element depth profile from EDS line scan of the metallic precursor after preheating process (sample A‐1‐h). c) The calculated X‐rays penetration depth of Cu‐Sn‐Zn alloy for different grazing incidence angles. The component of Cu‐Sn‐Zn alloy is Cu‐poor and Zn‐rich (Cu/Zn+Sn = 0.8, Zn/Sn = 1.2). d) XRD patterns of sample A‐1‐h with different penetration depths.

To better understand the formation mechanism of double layer distribution, the diffusion of Zn element in the metallic precursors after each different annealing processes was studied. Cross‐section morphology and element depth profile of the metallic precursor after preheating process (sample A‐1‐h) are shown in **Figure** **2**. Sample A‐1‐h is divided into two layers, as shown in Figure 2a. EDS depth profile (Figure 2b) shows that the content of Zn element in the bottom layer of the film is much higher than that in the top layer of the film. Oxygen is also detected, which is reasonable as considering the solution environment in electrodeposition process. Figure 2c shows the calculated X‐rays penetration depth of Cu‐Sn‐Zn alloy for different grazing incidence angles according to the equation^13^

(1) $$z=\frac{1}{\mu }{\left(\frac{1}{\sin\alpha_{i}}+\frac{1}{\sin(2\theta -\alpha_{i})}\right)}^{\;-1}$$

where *z* is the penetration depth, α_i_ is the incident angle, 2θ is the Bragg angle, and μ is the attenuation coefficient. The penetration depths are 270, 670, 1330, and 1980 nm as the incident angles are 0.2°, 0.5°, 1°, and 1.5°, respectively. XRD patterns of sample A‐1‐h with these different penetration depths are shown in Figure 2d. As penetration depth is 270 nm, only Sn and Cu_6_Sn_5_ phases are detected. Diffraction peaks of Cu_5_Zn_8_ coincide with the diffraction peaks of Cu_6_Sn_5_ at 43.1° and 62.7°, but there is no other diffraction peak of Cu_5_Zn_8_ phase appeared in XRD pattern, which indicates that the diffraction peaks at 43.1° and 62.7° are mainly contributed to Cu_6_Sn_5_. Mo reflection at 40.6° is cut off because of its too high intensity, so 72.6° is chosen to illustrate the influence of penetration depth. Diffraction peaks of phases containing Zn element (ZnO, Cu_5_Zn_8_) and Mo start to appear when the penetration depth increases to 670 and 1330 nm, respectively. This is consistent with the observed scanning electron microscopy (SEM) image (Figure 2a). On the basis of these results, we conclude that Zn element aggregates at the bottom of the film after preheating process.

**Figure 3:**
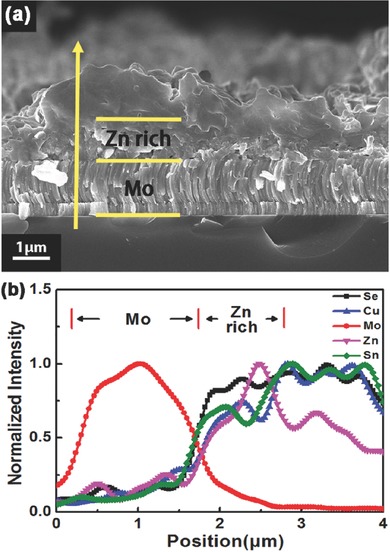
a) Cross‐section SEM image and b) EDS depth profile of sample A‐1‐hs after long time annealing process at 300 °C.

Figure S2 (Supporting Information) presents the XRD spectra of sample A‐1‐hs. After preheating process and preselenizing process, the pattern shows that the film only contains binary selenides. To better understand the diffusion and reaction of these binary selenides before being fully converted into CZTSe phase, A‐1‐hs samples were annealed at 200, 300, and 400 °C under N_2_ (10^4^ Pa) and Se (15 Pa) mixed atmosphere for a long time (named long time annealing process). XRD patterns of these thin films after long time annealing process (9 h) are shown in Figure S3 (Supporting Information). Obviously, the films only contain binary selenides after annealing at 200 and 300 °C. However, the diffraction peaks of CZTSe thin film at 17.4°, 28.35°, and 72.4° are clearly detected and most binary selenides peaks are disappeared as annealed at 400 °C, illustrating that the binary selenides are converted into CZTSe mostly. So 300 °C is chosen as the annealing temperature to investigate the diffusion of the binary selenides in the case of sufficient diffusion time. **Figure** **3** shows the cross‐section SEM image and EDS depth profile of sample A‐1‐hs after long‐time annealing process (9 h) at 300 °C. It is evident that Zn element is concentrated in the bottom of the film even after a long period of heat treatment. The contents of Cu and Sn elements in the bottom of the film are less than those in the upper layer of the film. In the Zn‐rich metallic precursor, it is therefore reasonable to deduce that the plenty of the ZnSe phase at the bottom cannot be fully reacted with Cu‐Sn‐Se phases when annealed at high temperature (550 °C), which leads to the phenomenon of double layer distribution. Toyama et al. used a sintered CZTS 4 in. disk target as the sputtering source.^14^ ZnSe phase is uniformly distributed in the film and does not concentrate on the bottom of the film. So the ZnSe can be fully reacted with other secondary phases. As a result, it does not have the phenomenon of double layer distribution, which demonstrate that the plenty of ZnSe phase at the bottom of the film is the reason of double layer distribution.

**Figure 4:**
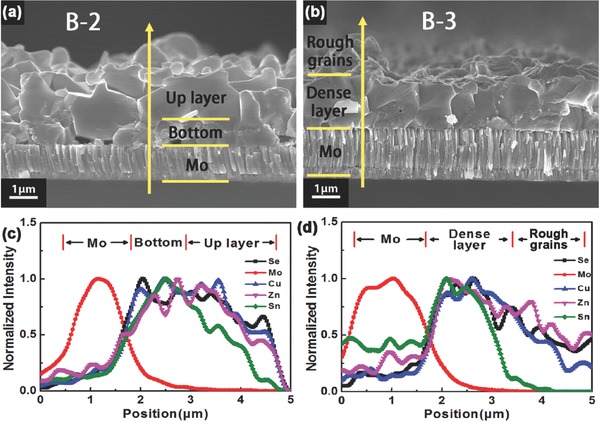
The cross‐section morphologies and element depth profiles from EDS line scan of sample a,c) B‐2 and b,d) B‐3. The up layer in (b) and (d) is the raised portion on the surface of sample B‐2.

After making clear the reason why double layer distribution appears, we try to eliminate this phenomenon. To make ZnSe phase reactive sufficiently, metallic precursors Cu/Sn/Zn were electrodeposited with various Zn/Sn ratios: 1.0 for sample A‐2 and 0.78 for sample A‐3. The compositions of these samples are summarized in Table 1. Compared with sample A‐1, only the content of Sn element is increased for samples A‐2 and A‐3. Samples B‐2 and B‐3 were fabricated from samples A‐2 and A‐3, respectively, using the same synthesis processes as sample B‐1. Obviously, samples B‐1, B‐2, and B‐3 are all Cu‐poor and Zn‐rich because of the loss of Sn element during selenization process at low pressure, though the compositions of metal elements in samples A‐1, A‐2, and A‐3 are quite different. The cross‐section morphologies of sample B‐2 and B‐3 are shown in **Figure** **4**a,b, respectively. The phenomenon of double layer distribution is reduced a lot in sample B‐2 compared with that in sample B‐1 (Figure 1a). And the grains next to the Mo back contact are much larger than those in sample B‐1. But there are still some voids at the bottom of the film. In sample B‐3, however, crystallization is dense and runs through the entire film without voids. The double layer distribution is completely disappeared. It illustrates that the excessive Sn element produces excessive SnSe_2_ phase during preselenizing process besides the suppression of the decomposition of CZTSe by Sn‐based vapor to some extent, which can make the plenty of ZnSe phase at bottom of the film to be fully reacted. Therefore, the phenomenon of double layer distribution gradually decreased with the increase of Sn content in the metallic precursor and disappeared finally. Especially, no MoSe_2_ layer is detected in both samples B‐2 and B‐3.

**Figure 5:**
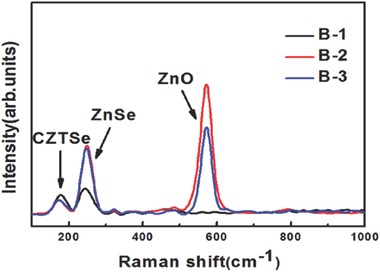
Raman spectra measured on the front surfaces of sample B‐1, B‐2, and B‐3. 325 nm laser beam is used as the exciting source.

EDS depth profile of sample B‐2 shown in Figure 4c indicates that all the elements contents gradually decrease from bottom to top in the up layer, which can be attributed to the rough surface morphology. It should be noted that the decrease of Sn element is greater than other elements in up layer. As Sn content in the precursor is increased further, that is the Zn/Sn ratio is decreased from 1.0 for sample A‐2 to 0.78 for sample A‐3, as shown in Figure 4d, Sn element is disappeared completely close to the top surface of sample B‐3. This indicates that the excessive Sn element in the metallic precursors is lost during the low‐pressure selenization process and the CZTSe phase in the top region of the films may have a decomposition reaction. **Figure** **5** shows the Raman spectra (excited at 325 nm) which were measured on the front surfaces of samples B‐1, B‐2, and B‐3. The ZnSe peak at 252 cm^−1^ can be observed in all samples. And the intensity of ZnSe peak of sample B‐1 is much lower than that of samples B‐2 and B‐3. ZnO peak at 575 cm^−1^ cannot be detected for sample B‐1, which illustrates that there is only little amount of ZnSe on the surface of sample B‐1. To make clear whether there are other secondary phases or not, the surface of all three samples are further measured by Raman scattering excited at 532 nm. The results are shown in Figure S4 (Supporting Information). The ZnO peak is detected in all samples,^15^ which indicates that all the samples contain ZnO phase in the top surface, but the content of ZnO is very low. No Cu_2_Se phase, SnSe phase, and Cu_2_SnSe_3_ phase are detected in all samples.^16^ Thus, decreasing the Zn/Sn ratios of samples A‐2 and A‐3 can lead to the easy decomposition of CZTSe phase on the surface of the films based on the EDS depth profiling and Raman spectra of samples B‐1, B‐2, and B‐3.

Based on the XRD and Raman spectra, a decomposition process of the surface of CZTSe film is suggested as below^17^

(2) $${\mathrm{Cu}}_{2}{\mathrm{SnZnSe}}_{4}\left(s\right)⇌{\mathrm{Cu}}_{2}\mathrm{Se}\left(I\right)+\mathrm{SnSe}\left(s\right)+\mathrm{ZnSe}\left(s\right)+\frac{1}{2}{\mathrm{Se}}_{2}\left(g\right)$$

(3) $$\mathrm{SnSe}\;\left(s\right)\;⇌\;\mathrm{SnSe}\;\left(g\right)$$

Beginning from the CZTSe surface, the continuous evaporation of SnSe gas phase from the films causes the continuous decomposition of the films. Thus, lots of voids are formed as SnSe evaporation and Cu_2_Se can easily diffuse from surface to the interior of CZTSe film along such voids during the decomposition process. When annealing at 550 °C, Cu_2_Se phase is liquid.^18^ The liquid Cu_2_Se phase will assist the grain growth as it goes through the film for a long time when the decomposition on the surface of the film lasts longer. And the SnSe*_x_* vapor supplied from SnSe*_x_* powders can suppress the decomposition of CZTSe and avoid the excessive loss of Sn element,^17^ which make samples B‐1, B‐2, and B‐3 have similar compositions (as shown in Table 1). It illustrates that the decomposition process on the surface of the CZTSe sample prepared with high Sn content precursor can last for a long time. As a consequence, the crystallization becomes better for samples B‐2 and B‐3 while the Zn/Sn ratio is decreased. The Zn/Sn ratio is 0.78 for sample A‐3, that is, the content of Sn is very high and can afford much Sn for decomposition and afford sufficient liquid Cu_2_Se phase for the crystallization. So the crystalline quality of sample B‐3 is much better than other samples. Therefore, it is available both from the elimination of double layer distribution and high crystalline quality through increasing the Sn content in the metallic precursor and annealing at low pressure under Se and SnSe*_x_* mixed atmosphere.

### 2.2 The Influence of Double Layer Distribution on the Performance of CZTSe Solar Cells

**Figure 6:**
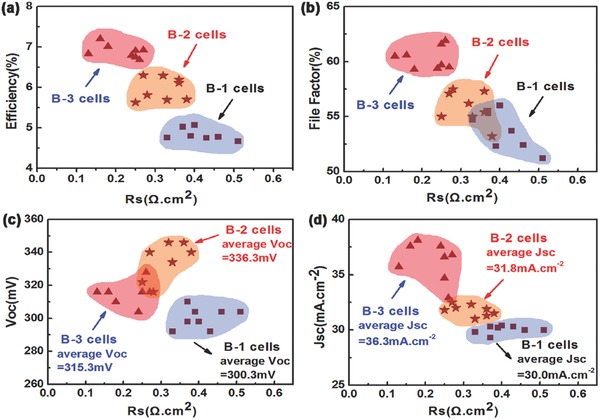
The performances statistics (*R*_s_, Eff, FF, *V*_oc_, and *J*_sc_) of three group thin film solar cells, which are fabricated with the same processes based on sample B‐1, B‐2, and B‐3, respectively. Each group contains eight solar cells. All of the *R*_s_ are fitted with the light *J*–*V* curve using the equation in ref. 19.

Three groups thin film solar cells were fabricated based on samples B‐1, B‐2, and B‐3 (named as cell B‐1, cell B‐2, and cell B‐3, respectively). The statistics performances of eight solar cells of each groups are presented in Table S1 (Supporting Information). The series resistance (*R*_s_) resolved efficiency (Eff), fill factor (FF), open circuit voltage (*V*_oc_), and short circuit current (*J*_sc_) of three groups of solar cells are shown in **Figure** **6**. A linear fit to the data based on light current density–voltage (*J*–*V*) curve gives an intercept of *R*_s_ from the equation^19^

(4) $$\frac{dV}{dJ}=R+\frac{AkT}{q}{\left(J+J_{L}-GV\right)}^{-1}$$

where *A*, *J*_L_, and *G* are the diode ideality factor, photocurrent, and shunt conductance, respectively. Compared with the group of cell B‐1 and cell B‐2, cell B‐3 shows a much better performance. The *R*_s_ of the group of cell B‐3 ranges from 0.13 to 0.27 Ω cm^2^, which can be attributed to the improved interface contact between CZTSe film and Mo substrate. As a result, the average *J*_sc_ is increased to 36.3 mA cm^−2^. And the FF and Eff of these solar cells range from 59.3 to 61.9% and 6.68 to 7.2%, respectively. The average *V*_oc_ of B‐3 cells is about 315.3 mV, lower than that of B‐2 cells.

**Figure 7:**
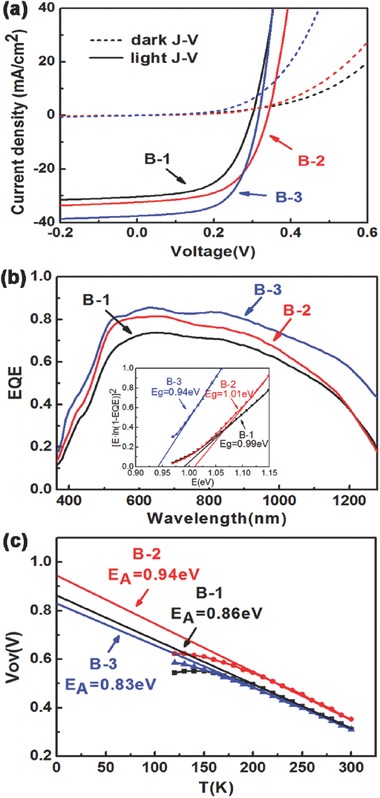
The a) light *J*–*V* curves, b) EQE responses, and c) the temperature‐dependent *V*_oc_ of the champion solar cell B‐1, B‐2, and B‐3, respectively. Inset of (b) *E*_g_ of these three thin film solar cells based on EQE response.

The comparison of dark/light *J*–*V* characteristics and spectral photoresponse (external quantum efficiency, EQE) of the champion solar cells in each groups of cells B‐1, B‐2, and B‐3 are shown in **Figure** **7**a,b, respectively. And the corresponding solar cell parameters are summarized in **Table** **2**. The performance improvement of champion cell B‐3 is mainly relying on the enhanced *J*_sc_ and decreased *R*_s_. The EQE response of champion cell B‐3 is higher than that of champion cell B‐1 and champion cell B‐2 in the whole wavelength region, which indicates the superiority of the excellent crystalline quality. However, the EQE response of cell B‐3 in the region of wavelength larger than 1100 nm is higher than others, which indicates that the band tail states are high in cell B‐3. The energy bandgaps (*E*_g_) are 0.99, 1.01, and 0.94 eV for cells B‐1, B‐2, and B‐3, respectively, determined from EQE data (inset of Figure 7b). The temperature‐dependent *V*_oc_ of these cells are shown in Figure 7c. The activation energy (*E*_A_) can be obtained when the linear extrapolation to *T* = 0 K. It should be pointed out that the *E*_A_ of cells B‐1 and B‐3 (0.86 and 0.83 eV) are much lower than the *E*_g_ determined from EQE data (0.99 and 0.94 eV). However, the *E*_g_ and *E*_A_ are very close to 1 eV for cell B‐2. This indicates that there exist more additional recombination process in cells B‐1 and B‐3, which can be considered as the reason of the double layer distribution in the CZTSe/Mo interface of cell B‐1 and why the average *V*_oc_ of B‐3 cells is lower than that of B‐2 cells.

**Table 2:** List of three champion performance solar cells fabricated with sample B‐1, B‐2, and B‐3. The *R*_s_ and *A* are fitted based on dark *J*–*V* curves using the equation in ref. 19

Solar cell	η [%]	*V*_oc_ [mV]	*J*_sc_ [mA cm^−2^]	FF [%]	*R*_s_ [Ω cm^2^]	*A*
B‐1	5.07	298	30.4	56.0	0.54	1.74
B‐2	6.30	340	32.5	57.1	0.51	1.98
B‐3	7.20	316	37.6	60.6	0.14	1.54

**Figure 8:**
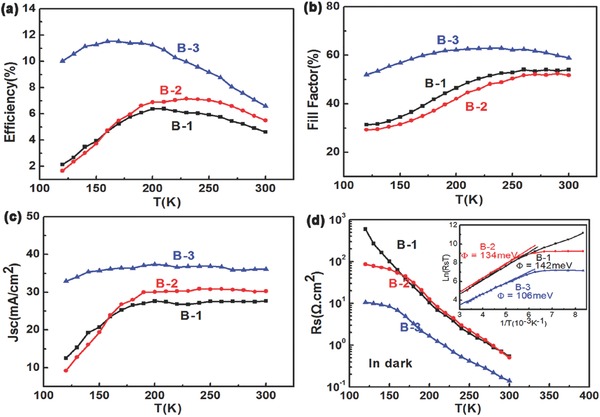
Temperature‐dependent efficiency, FF, *J*_sc_, and *R*_s_ of the champion solar cell B‐1, B‐2, and B‐3, respectively. The *R*_s_ are fitted based on dark *J*–*V* curves. Inset of (d) is the calculated blocking contact barrier height of cell B‐1, B‐2, and B‐3.

The temperature‐dependent photovoltaic performances of cells B‐1, B‐2, and B‐3 are shown in **Figure** **8**, respectively. The efficiency, FF, and *J*_sc_ of cells B‐1 and B‐2 are decreased dramatically as the temperature is lower than 180 K. This phenomenon is ascribed to the increase of the series resistance. We observe that the series resistance of all cells increases almost two to three magnitude from 300 to 120 K, which should be mainly attributed to the carrier freeze‐out effect because of the lack of a shallow accepter in CZTSe.^20^ However, *R*_s_ of cell B‐3 is much lower than that of cells B‐1 and B‐2 at the same temperature. The blocking contact barrier height (inset of Figure 8d) is calculated according to the equation^21^

(5) $$R_{s}\;=\;\frac{k}{qA*T}\exp\left(\Phi_{B}/kT\right)$$

where A* and Φ_B_ are the effective Richardson constant and barrier height. The obtained blocking contact barrier height is 142, 134, and 106 meV for cells B‐1, B‐2, and B‐3, respectively. Obviously, Φ_B_ of cell B‐3 is lower than others, confirming the excellent back interface contact of cell B‐3.The highest *R*_s_ of cell B‐3 is about 10 Ω cm^2^, lower than that of cells B‐1 and B‐2, respectively, partly because of the remarkable back interface contact of cell B‐3. An interesting finding is that the Eff, FF, and *J*_sc_ of cells B‐1 and B‐2 begin to have a significant reduction only when the *R*_s_ exceeds 20 Ω cm^2^ at 180 K. That is why, although the *R*_s_ of cell B‐3 increases with decreasing temperature, the performances of the solar cell do not have significant deficit as the temperature decrease. Note that the series resistance of cell B‐2 and B‐3 does not increase indefinitely as the temperature is lower than 150 K, which can be attributed to a finite leakage resistance *R*_L_ across the back contact diode and a better contact.

## 3 Conclusion

In this study, CZTSe solar cells were fabricated with electrodeposited metallic precursors and annealed at low pressure under Se and SnSe*_x_* mixed atmosphere. The phenomenon of double layer distribution was completely eliminated through increasing the reaction probability of ZnSe with Cu‐Sn‐Se phases by increasing Sn content in the metallic precursors. At the same time, the formation of MoSe_2_ was suppressed successfully. The quality of the crystal got a great improvement because of the assisted growth of CZTSe grains by liquid Cu_2_Se. Thus, the back contact between CZTSe absorber and Mo substrate was improved significantly. The series resistance of the solar cells reduced to 0.14 Ω cm^2^ and the *J*_sc_ raised to 37.6 mA cm^−2^. Although a large number of binary selenide (ZnSe) was introduced on the surface of the film, a CZTSe solar cell with efficiency of 7.2% was fabricated.

This work opens a novel route to eliminate the phenomenon of double layer distribution of CZTSe film prepared by selenization method, thus the back interface contact is improved further. The method should also be effective to CZTS and CZTSSe thin films, which presents a wide range of possibilities for the development of high‐efficiency thin film solar cells.

## 4 Experimental Section

*Preparation of Films and Solar Cells*: Metallic Cu/Sn/Zn thin films were sequentially electrodeposited onto Mo‐coated soda‐lime glass substrate according to the method reported in ref. 6. The preparation processes of CZTSe are shown in Figure S1 (Supporting Information). Before the precursors were annealed at the temperature from 250 to 350 °C for 26 min under Se (15 Pa) atmosphere to fully convert metallic alloy into binary phases (named preselenizing process), the as‐electrodeposited metallic stack precursors were first preheated at 250 °C for 120 min under N_2_ (10^4^ Pa) atmosphere (named preheating process) to improve the quality of the final CZTSe film. Then the samples were annealed at 550 °C for 20 min under Se (70 Pa) and SnSe*_x_* (1 Pa) mixed atmosphere (named low‐pressure preselenization process). The heating rate of the substrates is about 40 °C min^−1^. Se and SnSe*_x_* vapors were supplied by heating solid Se pellets and SnSe*_x_* powders, respectively. Then, the CdS buffer layer with thickness of 50 nm (via chemical bath deposition), 50/450 nm i‐ZnO/Al‐ZnO (via RF sputtering), and 50 nm/2 µm Ni/Al metal grid (via thermal evaporation) were deposited separately to fabricate thin‐film solar cells.

*Characterization*: The compositions of the films were determined by MagixPW2403 X‐ray fluorescent (XRF) spectrometer with an Rh‐anode, which was calibrated by inductively coupled plasma spectroscopy. The structural properties of the precursors were measured using a Philips X‐pert Pro X‐ray diffractometer with Cu radiation. A scanning electron microscope (SEM, JEOL JSM‐6700) coupled with an EDS was used to analyze the cross‐sectional morphology and depth profiles of CZTSe films. A Renishaw inVia Raman spectroscopy was used to analyze the phase of the CZTSe samples, using 325 and 532 nm laser as the exciting source. The current density–voltage (*J*–*V*) characteristics of the CZTSe solar cells were measured by a solar simulator under illumination by a standard AM 1.5 G spectrum of 1000 W m^−2^ at room temperature, which was calibrated with a standard monocrystalline Si reference solar cell. EQE measurements of the cells were performed by measuring the short‐circuit current with spectrally resolved monochromatic light.

## Conflict of Interest

The authors declare no conflict of interest.

## Supporting information

Supplementary
