# Supplementary material for: Modified Back Contact Interface of CZTSe Thin Film Solar Cells: Elimination of Double Layer Distribution in Absorber Layer
Source: Adv Sci (Weinh). 2017 Nov 20;5(2):1700645. doi: 10.1002/advs.201700645 (PMC5827099; doi:10.1002/advs.201700645)
Supplement: Supplementary file 1 — Supplementary [file ADVS-5-1700645-s001.pdf]

Copyright WILEY-VCH Verlag GmbH & Co. KGaA, 69469 Weinheim, Germany, 2017.

ADVANCED SCIENCE
Open Access

Supporting Information

for *Adv. Sci.,* DOI: 10.1002/advs.201700645

Modified Back Contact Interface of CZTSe Thin Film Solar Cells: Elimination of Double Layer Distribution in Absorber Layer

Zhaojing Zhang, Liyong Yao, Yi Zhang, Jianping Ao, Jinlian Bi, Shoushuai Gao, Qing Gao, Ming-Jer Jeng, Guozhong Sun, Zhiqiang Zhou, Qing He, and Yun Sun*

Copyright WILEY-VCH Verlag GmbH & Co. KGaA, 69469 Weinheim, Germany, 2016.

Supporting Information

Modified Back Contact Interface of CZTSe Thin Film Solar Cell: Elimination of Double Layer Distribution in Absorber Layer

Zhaojing Zhang, Liyong Yao, Yi Zhang, Jianping Ao*, Jinlian Bi, Shoushuai Gao, Qing Gao, Ming-Jer Jeng, Guozhong Sun, Zhiqiang Zhou, Qing He, Yun Sun*

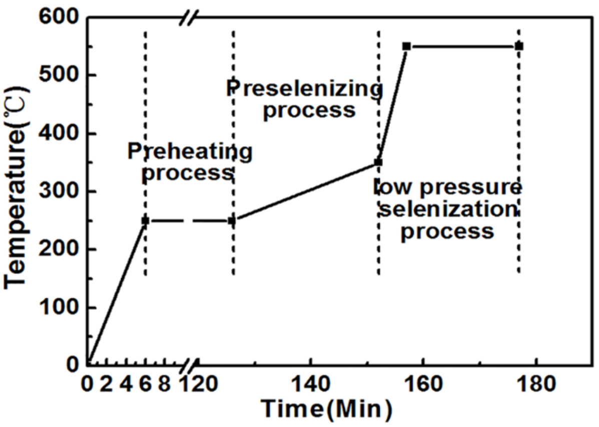

Figure S1. Temperature profiles of the annealing process for Cu/Sn/Zn metallic precursor.

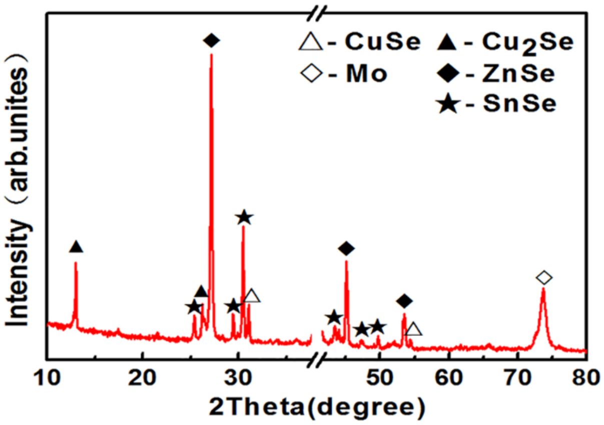

Figure S2. XRD pattern of the precursor after preselenizing process (sample A-1-hs).

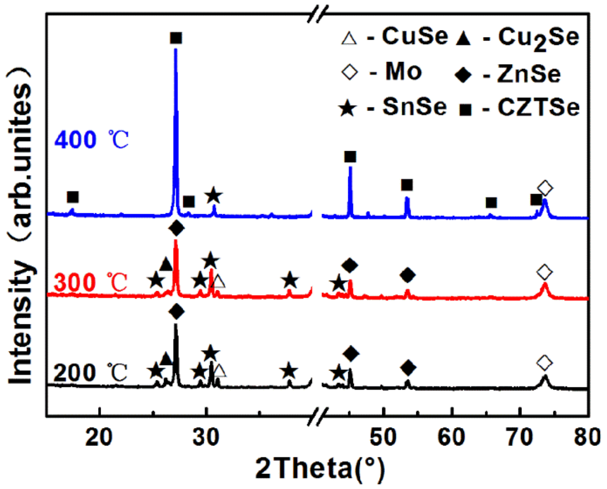

Figure S3. XRD patterns of sample A-1-hs annealed at 200 °C, 300 °C, and 400 °C for 9 hours.

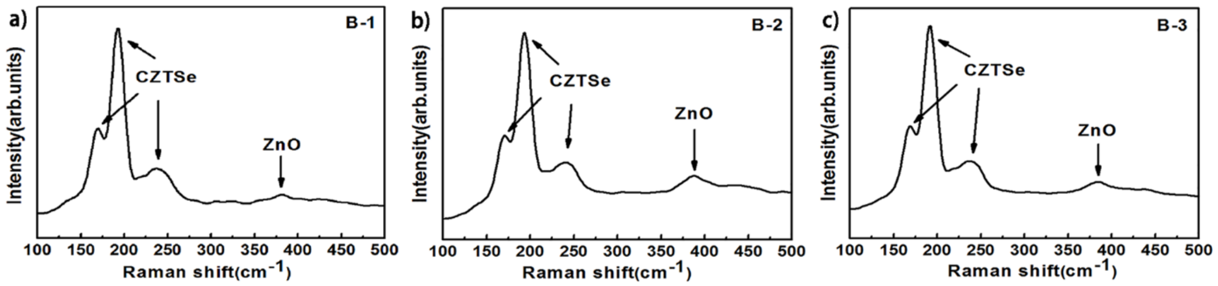

Figure S4. Raman spectra measured on the front surfaces of sample B-1, B-2, and B-3, recorded by exciting the samples with 532 nm laser beam.

Table S1. Performances of $Cu_2ZnSnSe_4$ (CZTSe) solar cells based on sample B-1, B-2, and B-3.

**cells**	**Cell number**	**V_{oc} [mV]**	**J_{sc} [mA cm^{-2}]**	**FF [%]**	**Eff [%]**
**B-1**	**#1**	**298**	**30.4**	**56**	**5.07**
	#2	292	29.8	54.7	4.76
	#3	298	30.3	55.5	5.02
	#4	292	30.3	53.7	4.75
	#5	310	29.3	55.3	5.03
	#6	304	30	52.4	4.77
	#7	304	30.2	52.3	4.8
	#8	304	30	51.2	4.67
**B-2**	**#1**	**340**	**32.5**	**57.1**	**6.3**
	#2	346	32.3	56.2	6.29
	#3	340	31.5	53.2	5.7
	#4	346	31.3	57.3	6.19
	#5	346	31.9	55.4	6.11
	#6	316	32	57.5	5.81
	#7	334	31	55	5.69
	#8	322	31.8	55	5.63
**B-3**	**#1**	**316**	**37.6**	**60.6**	**7.2**
	#2	310	38.1	59.3	7.01
	#3	328	32.9	61.9	6.68
	#4	316	34.7	61.6	6.75
	#5	316	36.6	59.7	6.91
	#6	316	36.8	59.5	6.92
	#7	304	37.6	59.4	6.79
	#8	316	35.7	60.5	6.83
